# The role of women in learning games and water management outcomes

**DOI:** 10.1093/pnasnexus/pgaf243

**Published:** 2025-07-31

**Authors:** Ivo Steimanis, Thomas Falk, Lara Bartels, Vishwambhar Duche, Björn Vollan

**Affiliations:** Chair for Sustainable Use of Natural Resources, School of Business and Economics, University of Marburg, Am Plan 2, Marburg 35032, Germany; Natural Resources and Resilience Unit, International Food Policy Research Institute, 1201 Eye St., NW, Washington, DC 20005-3915, USA; Research on Collective Goods, Max Planck Institute, Kurt-Schumacher-Straße 10, Bonn 53113, Germany; Research Program on Innovation Systems for the Drylands, International Crops Research Institute for the Semi-Arid Tropics, Patancheru, Telangana 502324, India; Chair for Sustainable Use of Natural Resources, School of Business and Economics, University of Marburg, Am Plan 2, Marburg 35032, Germany

**Keywords:** water management, social dilemma, experiential learning, games, India

## Abstract

Economic games have emerged as promising tools for fostering sustainable resource management, yet their gender dynamics remain underexplored. We examine how women’s participation relates to the effectiveness of game-based learning in 56 Indian communities facing water management challenges. These structured experiential learning environments allow participants to develop system understanding, problem-solving capacities, and collective action through active engagement. Our results suggest that greater female involvement is associated with improved water management outcomes 2 years after the intervention. Notably, the presence of female leaders correlated with broader participation among women, which in turn was linked to the development of more effective management rules. These findings indicate that gender-balanced participation may enhance the success of such interventions. Incorporating women in game-based learning has the potential to support long-term improvements in resource management, highlighting the importance of inclusive approaches.

Economic games are emerging as cost-effective and scalable tools for experiential learning in sustainable natural resource management (1, 2). By immersing participants in interactive decision-making scenarios, these games facilitate knowledge acquisition through experience (3, 4). However, evidence on their long-term impact on real-world behavior remains limited (3, 5, 6).

To address this gap, we conducted economic games with dam users in 56 randomly selected villages in Madhya Pradesh, India. Despite the critical role of these dams in irrigation and domestic water supply, maintenance remains inconsistent, and water use lacks coordination. Communities that participated in the games showed significantly greater dam maintenance efforts 2 years later compared to 28 control communities (6).

This study specifically investigates the role of women’s participation and female leadership in shaping water management outcomes. India’s government-mandated quotas for women, low-caste community members, and ethnic minorities in village councils have been key to expanding access to public goods, such as drinking water (7, 8). By enabling women to express their preferences, hold leaders accountable, and engage in decision-making, such policies have improved governance (9, 10). We hypothesized that women’s involvement in game-based interventions, particularly as primary water users, would have similar positive effects on dam governance and maintenance. By navigating social dilemmas within the game and exploring solutions, women may be empowered to advocate for better water management in real life.

Our intervention is based on Janssen et al.’s irrigation game (11), which simulates key challenges in water management. Participants decide how much of their endowment to invest in dam maintenance and which crops to plant, influencing water availability and distribution. The game was followed by a guided group discussion to reflect on experiences and strategies. Each community’s participant composition varied, including an average of 0.4 female leaders, 3.4 nonleader women, 1.2 male leaders, and 9 nonleader men. Communities were categorized into four groups based on leadership composition: no leaders (n=9), only male leaders (n=27), only female leaders (n=12), and mixed-gender leadership (n=8). To assess long-term impact, surveys were conducted with key community informants 2 months before and 2 years after the games. These surveys measured the presence of dam management rules and whether the dam had been maintained in the past 12 months.

## Results

**Fig. 1.**
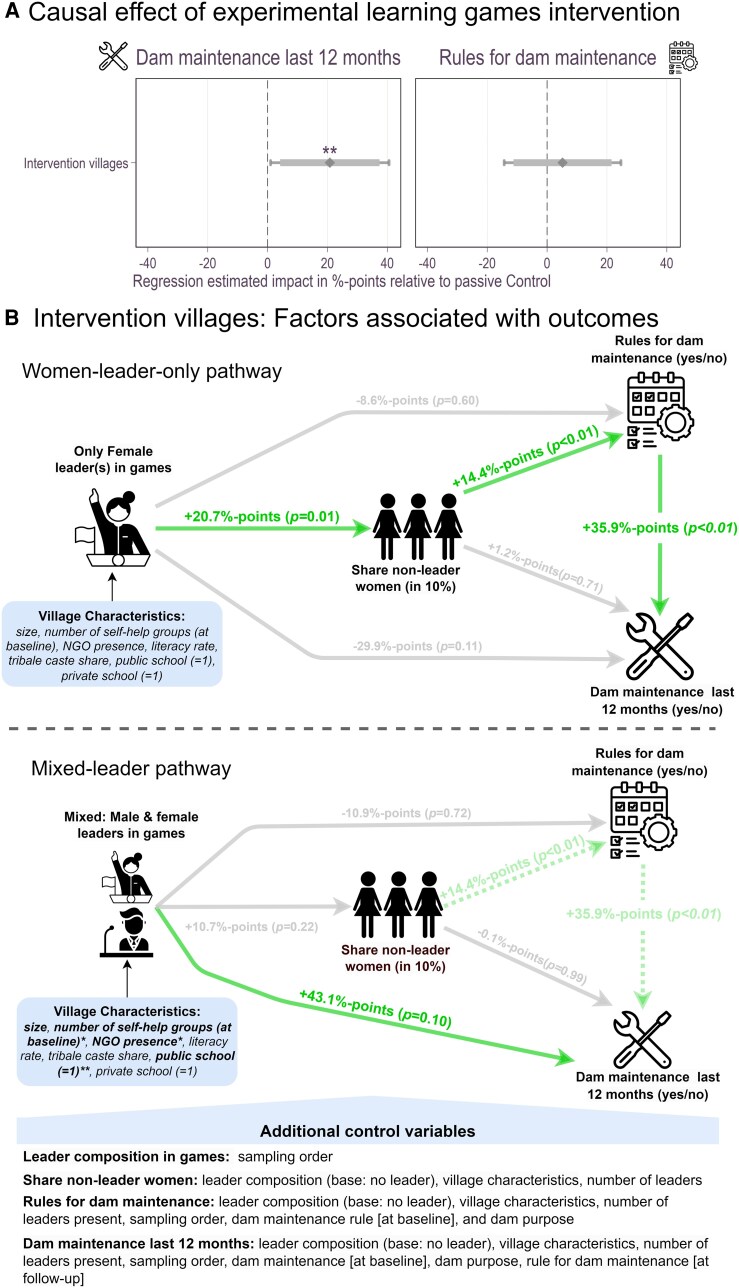
The impact of women’s participation in games on sustainable water management. A) The main intervention effect on dam maintenance (yes/no) and related rules (yes/no) with 90 and 95% CI using a Probit regression are showed. The estimations control for baseline level of the outcome variable, dam purpose, village size, NGO presence, number of SHGs, literacy rate, tribal caste share, number of public schools. We lose two observations due to missing values in these control variables. B) The pathway for villages with only female leader(s) present is visualized, while B) visualizes the pathway for villages with both male and female leaders participating. All effects are relative to villages where no leaders participated. Estimates are from a structural equation model with robust standard errors. See Supplementary material, Dataset S8 for the intervention effect estimates and Supplementary material, Dataset S5 for all SEM estimates.

Figure 1A shows that the intervention significantly increased dam maintenance by 20%-points (β=20.78, p=0.03, 95% CI=1.00,  40.57) but not rules for dam maintenance (β=5.17, p=0.60, 95% CI=−14.34,24.67) compared to control communities.

To explore factors associated with these outcomes, we now focus on intervention communities to analyze the relationship between leader participation, gender composition, and water management outcomes using a structural equation model (SEM). As leadership participation and gender composition were not randomly assigned, community-specific characteristics could influence both women’s participation in the learning games and collective action for dam maintenance. To mitigate such concerns, we include the presence of self-help groups, schools, and NGO activity as proxies for villages with stronger institutional support for gender inclusion, as these factors are often linked to women’s empowerment. Additionally, these characteristics may directly influence water management outcomes (see Materials and methods for details).

Communities differed in which types of leaders participated in the games, and we categorized them accordingly: no leaders, only male leaders, only female leaders, or mixed-gender leadership. In our analysis, we compare each group to villages without leaders. Communities with only male leaders did not show significant differences in women’s participation, rule creation, or dam maintenance relative to nonleader communities (see Supplementary material, Dataset S5) and are therefore not included in the figures or main discussion. This lack of difference suggests that leadership presence alone is not sufficient to explain the observed improvements in outcomes. The effects seen in female-only and mixed-gender leader communities are therefore more plausibly related to the gender composition of leadership rather than to leader participation per se.

The first pathway indicates the effect for communities where only female leaders participated in our games (Fig. 1B). In such situations, significantly more nonleader women joined the learning sessions compared to villages without leaders (β=20.72, P=0.01, 95% CI (CI)=4.76,36.69). A higher number of nonleader women in the games was associated with increased creation of dam maintenance rules (β=14.44, P<0.01, 95% CI=8.68,20.19), a crucial prerequisite for actual dam maintenance (β=35.86, P<0.01, 95% CI=13.81,57.91). Consequently, greater female participation is indirectly associated with improved dam maintenance (β=5.18, P<0.01, 95% CI=1.39,8.97). Notably, the presence of only female leaders was not significantly associated with measured village characteristics, suggesting that improvements in water management are more likely linked to the gender composition of the sessions rather than solely to preexisting village differences.

The second pathway examines how inclusive leadership participation influences water management outcomes. In villages with greater institutional support for gender inclusion—characterized by a higher number of female self-help groups, educational facilities, and NGO activities—mixed-gender leadership participation in the learning games was more likely (Fig. 1B). The presence of both male and female leaders improved dam maintenance compared to villages without leaders (β=43.06, P=0.10, 95% CI=−0.91,95.20), suggesting direct benefits of mixed-gender leadership participation in the learning sessions for water management. While mixed-gender leadership is directly associated with improvements in dam maintenance, it does not significantly increase women’s participation in the games (β=10.69, p=0.22, 95% CI=−6.38,27.76). By contrast, the presence of only female leaders strongly increased women’s participation, which is linked to greater rule creation and, ultimately, better water management outcomes.

## Discussion

Over the past decades in India, government policies, civil society efforts, and international aid organizations have invested heavily in women’s empowerment through self-help groups (12) and binding quotas (13). Women are often better organized than men (14) and play a crucial role in local governance. Our findings highlight that leadership composition influences the effectiveness of experiential learning interventions. Both female-only and mixed-gender leadership structures produced the best outcomes, reinforcing prior research on the role of leadership in self-governance (15), diversity (16), and collective action. Women’s participation may enhance prosocial values and introduce diverse perspectives (15), aligning with studies on the effectiveness of gender quotas in natural resource management (17).

These outcomes are potentially driven by mechanisms through which the games translated into broader community engagement and institutional change. In nearly half of the intervention villages (46%), the games were formally discussed in community or village council meetings—forums where new rules are often introduced or reinforced (6). Such discussions suggest that the experiential format not only engaged participants during the sessions but also triggered wider deliberation within local governance structures. While these conversations were not significantly more common in villages with female leaders, their overall prevalence supports the interpretation that the intervention helped initiate rule-making processes beyond the individual level. Supplementary material, Dataset S10 shows that while in-game behavior did not differ systematically by leadership type, higher nonleader female participation was associated with more positive player experiences and a greater likelihood that the game was discussed in formal village meetings, underscoring how broader gender inclusion may foster community-level deliberation

A back-of-the-envelope cost-benefit analysis suggests that implementing the intervention in one community costs ∼USD $75 and increased the likelihood of proper dam maintenance by 20%-points (see Fig. 1A). While the precise economic impact of this improvement requires further study, available regional estimates provide context for potential benefits. According to NGO Action for Social Advancement (18), fully functional dams in the region were estimated to generate on average about $1,965 in annual agricultural and fishing income compared to $689 for a partially functional dam (adjusted to 2022 prices). These estimates suggest that deterioration from full to partial functionality could represent potential annual losses of $826–1,516 per dam. Our model suggests that communities achieving balanced gender representation in the learning sessions may experience greater maintenance improvements. Based on these associations and the regional value estimates, the economic benefits could potentially exceed the intervention cost within the first year (see Supplementary material, Dataset S1 for calculation assumptions and limitations). These estimates should be interpreted cautiously, yet the intervention’s low implementation cost and ease of delivery have facilitated its integration into NGO and government programs, reaching more than 4,800 communities across India by 2023.

Despite these encouraging results, we caution against overinterpreting the point estimates from this study, which involved a relatively small number of villages and observational variation in leadership composition. The actual effects of the games and the roles that gender composition and leader participation play may be smaller when implemented at scale. Our design reflects how such interventions are typically implemented in practice: through locally appropriate recruitment channels which capture the interplay of leadership, social networks, and local norms. Although this approach limits our ability to isolate the causal impact of individual components (such as the games, debriefing sessions, or specific types of participants), it offers valuable insight into how such interventions function under real-world conditions. Our comparison with census data shows that participants were generally younger and more literate than the district average, which was intended to positively influence receptivity to the intervention. Nonetheless, even modest increases in impacts due to higher female participation could be meaningful given the cost-effectiveness and flexibility with which such games can be integrated into existing programs.

Future studies should build on these findings by testing the potential of games for experiential learning in larger samples and diverse contexts, and by experimentally varying key components—such as the gender composition of participants, the involvement of community leaders, and recruitment methods—to better identify the mechanisms driving changes in resource governance. Most likely, the observed effects reflect the interaction of multiple factors rather than a single cause. Comparing game-based interventions to active controls, such as standard information workshops, would help determine whether the experiential elements provide added value over more conventional approaches.

Our study provides evidence that experiential learning games can be a valuable complement to empowerment and natural resource management programs. While the observed improvements in water management may be influenced by multiple factors, our results suggest that female participation in these sessions plays an important role. These findings underscore the value of integrating women into such interventions—not only as a matter of equity but also as a potential driver of more effective and sustainable resource management.

## Materials and methods

### Study implementation

**Fig. 2.**
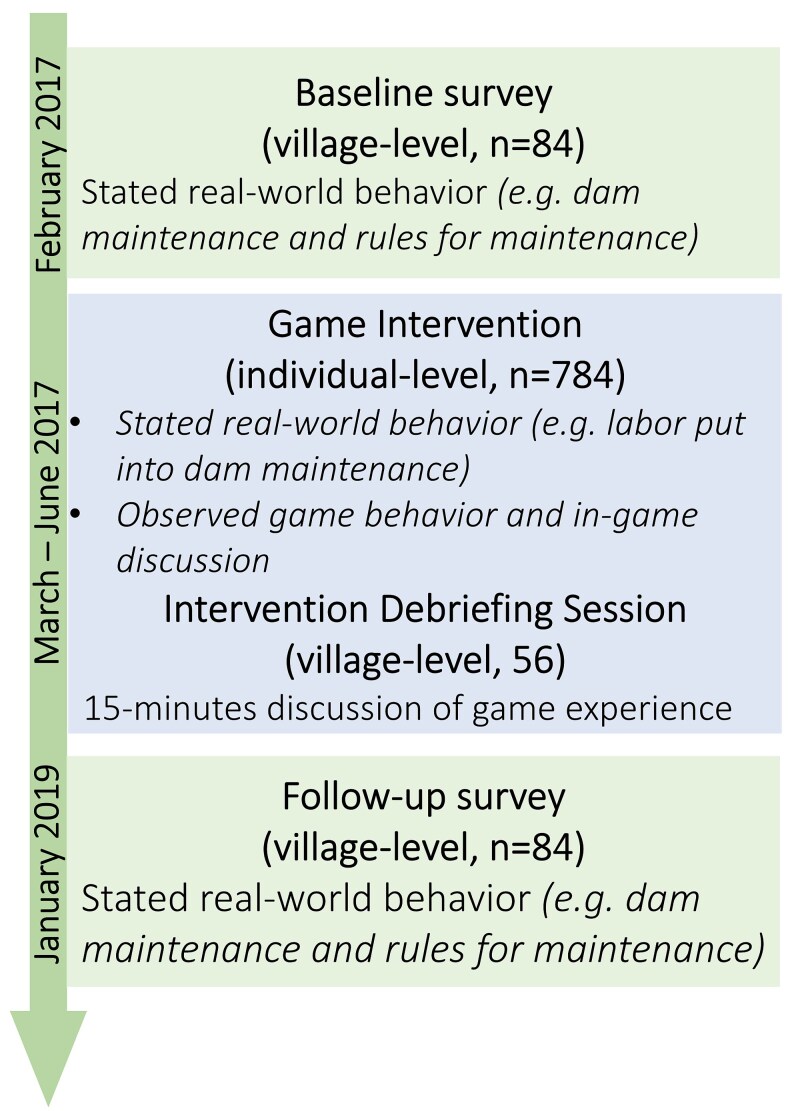
Overview of data sources used. The Supplementary material gives additional information on the participants in the games as well as an overview of the measurement of all outcome variables.

Based on a 2011 census village list, 56 study and 28 control communities were randomly selected. In intervention communities, we implemented the experiential learning intervention between baseline and follow-up surveys, while control communities received no intervention (Fig. 2). Local leaders and local experts were interviewed about villages’ water management and governance practices. These surveys provide our two main outcomes, which we use to measure behavioral change: (i) maintenance of the village dam in the last year and (ii) the existence of dam management rules. An overview of village-level survey measures collected at baseline and follow-up is available in Supplementary material, Dataset S4.

The learning sessions were conducted between March and June 2017 by Marburg University in collaboration with researchers from the International Crops Research Institute for the Semi-Arid Tropics (ICRISAT) and the Foundation for Ecological Security (FES, India). Although FES was involved in the broader research collaboration, the project was presented to participants as academic research with no relation to FES. A week prior to each session, a field facilitator met with a village council (panchayat) member to introduce the study and request the participation of 14 community members who were either direct users of the village dam or involved in local natural resource governance. Recruitment followed standard practices for community mobilization in the region and included an explicit request to ensure the participation of women, which was typically facilitated through female panchayat members. Leaders were defined as individuals holding formal or semiformal positions in village governance or natural resource management. This included elected panchayat members (n=36), individuals involved in community-based natural resource management (CBNRM) bodies (n=43), and leaders of self-help groups (SHGs, n=7). Three leaders also held informal leadership roles within cultural or community groups. The composition of leaders across these categories, and by gender, is detailed in the Supplementary material, Dataset S9.

On the day of the learning session, the lead facilitator first provided information about the project, the project partners, and the game, and introduced the facilitation team. S/he then explained the type of data to be collected, and the way the data would be stored and used. Expectations regarding the project outcomes were clarified. The facilitator informed the players again about the time required to run a session. After all these explanations, oral consent was obtained and a short survey was conducted before the learning session began. On average, participants were 33 years old, female participation was about 25%, about half had only primary education, and less than 5% were illiterate. Summary statistics for these characteristics are provided in Supplementary material, Dataset S2.

### Statistical analyses

We used a SEM to assess both direct and indirect pathways through which female leadership and participation impacted water management outcomes. SEM’s ability to model latent variables and test complex causal pathways makes it well-suited for disentangling the interplay between gendered participation, leadership, and institutional outcomes. All SEM analyses were conducted using STATA software with the “sem” and “gsem” commands.

To account for nonrandomized leadership participation, we control for village characteristics such as the number of female self-help groups, school presence, NGO activity, literacy rate, and share of tribal caste members. These factors not only indicate institutional support for gender inclusion but may also directly influence water management outcomes. To address potential biases from preexisting differences between villages, we control for baseline levels of dam maintenance and management rules, ensuring observed effects are not confounded by initial conditions.

Over time, the facilitation team improved in encouraging women to participate in the games. In the first 28 communities, an average of 2.5 women joined each learning session, while in the second half, this increased to 4.3 women (T-Test diff.=−1.8, P<0.01). While balancing tests detected no structural differences between early and late villages (Supplementary material, Dataset S3), limited statistical power due to sample size may obscure subtle variations. Controlling for session order and baseline outcomes further reduces temporal confounding. Robustness checks using generalized SEM (Supplementary material, Dataset S6) and excluding baseline dam rules (Supplementary material, Dataset S7) confirmed consistency across model specifications.

## Supplementary Material

pgaf243_Supplementary_Data

## Data Availability

The data and analysis scripts are available on Zenodo (https://doi.org/10.5281/zenodo.8009659).
