# Supplementary material for: The role of women in learning games and water management outcomes
Source: PNAS Nexus. 2025 Jul 31;4(8):pgaf243. doi: 10.1093/pnasnexus/pgaf243 (PMC12344488; doi:10.1093/pnasnexus/pgaf243)
Supplement: pgaf243_Supplementary_Data [file pgaf243_supplementary_data.zip › PNASNEXUS-PNASNEXUS-2025-00323-TR-s01.docx]

**Supporting Information for**

The Role of Women in Learning Games and Water Management Outcomes

Ivo Steimanis^a,1^, Thomas Falk^b^, Lara Bartels^c^, Vishwambhar Duche^d^, Björn Vollan^a^

Ivo Steimanis

Email: [ivo.steimanis@staff.uni-marburg.de](mailto:ivo.steimanis@staff.uni-marburg.de)

**This PDF file includes:**

Supporting text

Legends for Datasets S1 to S10

SI References

**Other supporting materials for this manuscript include the following:**

Datasets S1 to S10

Economic game design

The game was designed along the lines of other irrigation games, with a provision decision and an appropriation decision under asymmetric resource access (1). The game was played simultaneously with two groups of seven players each in 56 randomly selected villages in the Mandla district in Madhya Pradesh, India. Each group played a maximum of 10 rounds. The rounds were divided in two phases, with five rounds in each phase.

In the first phase, players kept their group assignments, positions along the feeder channel, and their decisions secret. To maintain anonymity but allow players to keep track of the consequences of their investment and crop decisions, they were given a personal sheet with their round earnings after each round. Before the second phase, the groups and player positions were reshuffled to maintain the anonymity of phase one. In phase two, players knew who the group members were and their positions. They could communicate between game rounds and individual decisions were disclosed. Contributions, crop choices, and earnings of all players were written on a poster visible for all players. All these changes were announced before the second phase started. Each session closed with a debriefing that allowed all players and other community members to discuss the experience of the game for approximately 15 minutes. The facilitators asked open-ended questions intended to relate the game experience to the community’s real-life water challenges.

In each game round, players received an initial endowment of 3,000 play rupees and made two interlinked decisions. First, they had to decide how much of their endowment to invest in collective dam maintenance (and how much to keep). The accumulated individual contributions determined the total amount of water available as a function of diminishing returns.

Second, players had to choose between a water-intensive crop that yields more income and a water-efficient crop that yields less income. We implemented this by modelling two crop types with distinct economic returns and water requirements. The water-consumptive crop yields a net return of 15,000 INR per hectare and requires 5,000 m³ of water per hectare. In contrast, the water-efficient crop produces a lower net return of 13,000 INR per hectare but requires only 3,000 m³ of water per hectare, representing a 40% reduction in water usage with only a 13.3% reduction in economic return.

Their decisions affect the distribution of water among the group members, and thus players’ earnings. In both phases, to ensure that participants took their investment decisions independently, two envelopes were given to them: an orange one containing the initial endowment of 3,000 play rupees; and an empty red envelope. If the players wanted to invest money in dam maintenance, they transferred their chosen amount to the red envelope. The envelopes were then handed back to the field assistants, who calculated the total group investment for this round. For the crop decision, decision cards were also given to the players in a (white) envelope to assure anonymity.

By design, there is never enough water in the dam such that all players can grow the water-consumptive crop. If players choose the water-consumptive crop, the water is depleted quickly, and overall group earnings are less than if everybody in the group chose the water-efficient crop. If all players invest approximately three-fourths of their endowment in dam maintenance in the first decision, there is enough water such that all players can grow the water-efficient crop.

In addition, players are placed successively downstream from the dam along the feeder channels; players closest to the dam have preferential access to the water. For example, player 2 only takes water for irrigation after player 1; player 3 after player 2; and so on to player 7. How much each player can take depends on how much water was provided in step 1 and how much water was left by the upstream players.

A group of seven players would maximize their income if every player invested approximately three-fourths of the endowment and opted for the water-efficient crop. Any heterogeneous crop choice would result in a group income between the two functions. In fact, the game is set up so that if just one player chooses the water-intensive crop, the last player will not have enough water for either crop, regardless of how much he or she invested and how much the group contributed in total. Therefore, the maximum group income can be reached by jointly investing INR 14,000 and all choosing the water-efficient crop. However, players have incentive to deviate from this tactic and choose the water-intensive crop with the higher net returns. Additionally, the randomly allocated asymmetric positions may influence their decisions. Thus, the particularity of this game lies in the relationship of the investments collected for maintenance, the crop choice, and the sequential access to the water, simulating the fundamental dilemma of common property resource management. Interlinking these factors creates a complex impact chain that closely replicates the collective action problems around surface water management in the study region.

The exact wording and all materials that were used to explain the game are included in the replication package on zenodo (https://zenodo.org/records/8009659).

Study implementation

The study was carried out by a team from the University of Marburg (UoM), the International Crops Research Institute for the Semi-Arid Tropics (ICRISAT), and the Foundation for Ecological Security (FES). The team coordinated with respective panchayats and the watershed committees of the sites. The partners jointly developed the intervention tool. FES identified local field assistants for data collection. Data management and analysis were carried out jointly by the UoM and ICRISAT. The ethics committee of the School of Business and Economics of the University of Marburg has reviewed the research and approves the practices of the research.

Sample selection: Village level

The Bhichiya block of Mandla district of Madhya Pradesh, India, was selected for this study. The project initially covered 90 villages, which were randomly selected from a list of all villages in the Mandla district, without any stratification, from a list of the 194 villages in the Mandla district reported by the official 2011 census. Of these 90 sample villages, 60 were assigned to the treatment group and 30 were assigned to the control group. Our final sample included only 84 villages: 56 treated and 28 control sites. Three sites (ID 34, 218, and 243) were dropped because there was no water infrastructure, and two sites (ID 254 and 378) were dropped because baseline survey data were not complete. Another village (ID 252) was dropped because too few people showed up for the game session.

Survey

A key informant baseline survey was conducted at the community level. The survey collected information about local water use and local water governance. A codebook with exact wording of the survey items is included in the replication package.

Local community members were identified as enumerators in coordination with FES. They received a two-day training on the questionnaire and the data collection process. Data collection was conducted in February and March 2017. First, enumerators visited the sample communities to explain the purpose and background of the research to community leaders. They then invited knowledgeable people, such as village presidents, ward members, village committee members, members of the water users association, teachers, and self-help group members, for the interview.

A few days later, between two and six key informants participated in the interviews, which were conducted at the village panchayat office, a temple, or a community hall. The enumerators first gave a brief introduction of the project, the project partners, and themselves. Oral consent was obtained from the key informants before the interviews started.

A key informant follow-up survey was conducted at the community level. The survey again collected information about local water use and governance. The follow-up survey was conducted by ICRISAT staff only. Otherwise, the same process was followed as for the baseline survey.

While reported dam maintenance increased, the observed prevalence of formal maintenance rules declined from 32% at baseline to 14% at follow-up, see Supplementary Dataset S4. This drop was present in both treatment and control sites, suggesting that it was not intervention-driven but rather a result of differences in how the question was asked at baseline versus follow-up. In a more recent still unpublished study in three different states of India, data from habitation records suggests that between 2021 and 2024, approximately 15% of communities introduced new water rules and there was no evidence that existing rules were abolished. As such we consider it highly unlikely that during the study period such a substantial suspension of rules has happened. The measuring effect in our data affects all villages similarly, it does not introduce systematic bias in our SEM, which relies on endline data. The baseline measure is used only as a control variable to account for pre-existing differences. One should be cautious in interpreting the absolute decline in rules to governance changes within communities. To test the robustness of our findings, we also ran the main SEM without controlling for baseline rule existence (see Supplementary Dataset S7), and the results remained qualitatively unchanged. This confirms that our key conclusions are not driven by inconsistencies in the baseline measure.

Economic game

**Invitation**: After the baseline survey interview, the enumerators provided a brief explanation of the nature of the intervention. They then gave a letter of invitation to the village leaders in the treatment communities, requesting the community’s participation in the experimental game. The invitation contained some background information about the project and requested that the leaders should contact a FES project manager if the community wanted to participate in the intervention. All communities who were randomly selected as treatment sites confirmed their interest in participating in the project.

**Facilitation team:** The facilitation team consisted of a student from the University of Marburg, a research fellow and a research assistant from ICRISAT, and 9 community members who were trained in facilitating the game. Community facilitators were identified with the support of FES. Three of the 12 facilitators were women.

Training of community facilitators: Over multiple days, we developed the capacity of community facilitators to lead the community participants through the games. The game protocol and material were translated into Hindi. The game was explained step by step, and the role of each facilitation team member was worked out. The game was then played repeatedly, first within the facilitation team and then with college students who were invited to participate in test runs. After the classroom training, two game sessions were arranged in communities that were not part of the sample. Once the facilitation team was trained, they started conducting game sessions in the sample communities.

**Recruitment of participants:** One male member of the facilitation team (the same throughout the study and not a member of FES) was appointed to visit the treatment communities a week prior to the intervention. He met in person with the local village council (panchayat) president or member to explain the study background and purpose and fix a convenient time and day for playing the game. For participant recruitment, the facilitator requested the panchayat representative to invite 14 community members who own or manage land near a common dam, who benefit from the dam or play key roles in local natural resource management. This ensured that all 784 participants could relate the game to their real-life context. The field facilitator requested to encourage women, who actively participate in village-level committees, self-help groups, the panchayat, or work as teachers as well as women who play no dedicated role in the community, to join the session. Typically, this mobilization of women was done through female panchayat members.

**Implementation timeline:** One day before the game, one member of the facilitation team revisited the community and informed all invited players about their participation and how long the session would take. He also identified a venue, typically a temple, community meeting place, school, or town hall, in consultation with community members.

The facilitation team always arrived in the community one hour before the game session to set up banners and posters before the players gathered at the venue.

Once all players had gathered, the lead facilitator provided information about the project, the project partners, and the game, and introduced the facilitation team. S/he then explained the type of data to be collected, and the way the data would be stored and used. Expectations regarding the project outcomes were clarified. The facilitator informed the players again about the time required to run a session. After all these explanations, oral consent was obtained.

After this introduction, the lead facilitator explained the game narrative and rules. Test rounds were conducted before the facilitators led the players through the actual game.

**Debriefing**: At the end of the whole process, we asked players to give us anonymous feedback about their satisfaction with the experiential learning experience. We used two boxes that were placed where they could not be observed. The boxes had the following meanings:

Box: 1 Yes, the game is good and I would like to participate in such types of games in the future.

Box: 2 No, the game is not useful and I am not interested to participate in such kinds of games in the future.

Each player received a token and then voted, one at a time, by placing the token in the box of their choice.

**Payments**: After completing the game and the debriefing, the facilitators paid the players. To test whether individual payments affected players’ choices and learning, we randomly allocated half the sites to have individual payments based on players’ “earnings” in the game, and half the sites to receive a single lump sum paid to the community. Individual payments where made in a discrete manner. For more details on this aspect of our study, please see Bartels et al. (2).

Return on Investment Estimation for Game Intervention

We conduct a back-of-the-envelope cost-benefit analysis of our intervention. Acknowledging great variations between specific dams, the NGO Action for Social Advancement estimated in 2008 that the increase in irrigation water availability from a fully functional dam results on average in annual additional agricultural income of INR 91,080. A partially functional dam would increase income on average by INR 33,120. We converted these values to US dollars (exchange rate 1:75) and 2022 prices (inflation rate 156%). Thus, in 2022 prices, a fully functional dam would provide benefits of US$1,895 and a partially functional dam US$689. Based on expert advice, we added an annual fish-farming income of US$70 (if the dam is fully functional). FES has estimated the average annual costs of regular dam maintenance to be approximately US$450. Approximately 60 percent of this amount covers expenses of building materials and machinery. Two fifths of the costs are the community members’ labor input estimated according to the daily rates of the social employment program under the Mahatma Gandhi National Rural Employment Guarantee Act.

We assume that a recently constructed or rehabilitated dam would be at least partially functional even without maintenance. The annual loss from not maintaining the dam would therefore be US$ 1,895 + $70 – $689 – $450 = $826. After three years, not maintaining the dam would lead to its complete breakdown. The annual loss due to not maintaining the dam would then be $1,895 + $70 – $450 = $1,516.

Our analysis of the main treatment effect indicates that the intervention increased the probability that a dam is properly maintained by 20 pp compared with control sites where the game was not played (Supplementary Dataset S8). We make the conservative assumption that the effect of the intervention on collective maintenance activities declines by 10 pp over a period of five years, although it is also possible that a positive experience of successful dam maintenance increases its effect.

Using these assumptions, we estimated the return on investment (ROI) for our game intervention based on its effect on maintenance probability over five years. With an initial cost of $72.44 per participant, the intervention generated increasing cumulative returns when discounted at 10% annually. The ROI grew from 28% in Year 1 to 733% by Year 5, reflecting both the durability of behavior change and the increasing value of maintenance benefits (from $826.26 in Years 1-3 to $1,515.56 in Years 4-5). The probability effect gradually decreased from 20 to 10 percentage points over the five-year period.

Focusing on the treatment sites only, the results of our SEM presented in the main text indicate that increasing the participation of women in the game session by 10 pp increases the probability that maintenance will take place by about 6 pp. If no women participate in a session, the model finds a 58 percent probability that maintenance takes place. This probability rises to 88 percent if an equal number of men and women participate, that is, a 30 pp increase in maintenance probability. Based on these numbers, we calculated the return on investment of equal gender participation compared to men only sessions. The results indicate that including equal numbers of women and men in game sessions pays back more than twofold in the first year – assuming no additional costs of women’s participation. After five years, the return on investment is 1146 percent.

Detailed calculations are provided in the Supplementary Dataset S1.

2017 Baseline Survey

1. Has your stop dam received any maintenance within the last 12 month?

□ Yes □ No

1. If yes, please state briefly what kind of activities have taken place?

| □ Removing silt | □ Repairing the earth walls of the dams |
| --- | --- |
| □ Removing vegetation | □ Repairing the main wall of the dam |
| □ Repairing sluice gates | □ Other (please list in bullet points) |

1. Within the last 12 month, which groups or organizations contributed to the maintenance of the dam?
2. Within the last five years, which groups or organizations contributed to the maintenance of the dam?
3. Are there any rules related to the maintenance of the dam? Please describe them as detailed as possible,
4. Do you remember any traditional old rules for dam management around your place? Please describe them!
5. Please list all types of benefits anybody enjoys related to the dam!
6. Within the last 12 month, which individuals, groups or organizations received water from the dam? (Please name the households who used the dam water for irrigation.)

| individuals, groups or organizations | Purpose of water use |
| --- | --- |
|  |  |
|  |  |
|  |  |
|  |  |
|  |  |
|  |  |

1. Are there any rules related to the water extraction? Please describe them!
2. Have there been any conflicts around the dam within the last 5 years?

*In addition, it would be good if some engineer could give a rating of the dam condition and an estimate of the amount the specific dam can store when being well maintained (can be rough estimate).*

| *Capacity reduced due to siltation* | □ *Not* □ *very little* □ *little* □ *much* □ *very much* |
| --- | --- |
| *Capacity reduced due to overgrowing vegetation* | □ *Not* □ *very little* □ *little* □ *much* □ *very much* |
| *State of the earth walls* | □ *very good* □ good □ fair □ poor □ *very poor* |
| *State of the main wall* | □ *very good* □ good □ fair □ poor □ *very poor* |
| *State of the sluice gates* | □ *very good* □ good □ fair □ poor □ *very poor* |
| *State of the feeder channels* | □ *very good* □ good □ fair □ poor □ *very poor* |

2019 Follow-up Survey

| Community^[[1]](#footnote-1)^: | Community ID^[[2]](#footnote-2)^: |
| --- | --- |
| Date: | Interviewer: |

Interview partner:

| □ | Village leader/Panchayat | □ | Farmer/dam user |
| --- | --- | --- | --- |
| □ | Water User Association member | □ | Other (Specify) |

| Contact number of Interview partner | 1) |
| --- | --- |
|  | 2) |

1. What kind of common water resources available in your community?

| □ | Stop dam | □ | Pond | □ | Bori-Bunds | □ | Other (specify) |
| --- | --- | --- | --- | --- | --- | --- | --- |
|  |  |  |  |  |  |  |  |

1. Has your stop dam received any maintenance after March 2017?

| □ | Yes | □ | No |
| --- | --- | --- | --- |

1. If yes, please state briefly what kinds of activities have taken place?

| □ | Removing silt | □ | Repairing the earth walls of the dams |
| --- | --- | --- | --- |
| □ | Removing vegetation | □ | Repairing the main wall of the dam |
| □ | Repairing sluice gates | □ | Other (please list in bullet points) |

1. Which groups or organizations contributed to the maintenance of the dam? (Please tick the box and give detailed information)

| □ | Local Governance | □ | NGO |
| --- | --- | --- | --- |
| □ | Community | □ | Government |
| □ | Community based Organization |  |  |

1. Are there any rules prepared by the community members related to maintenance of dam?

| □ | Yes | □ | No |
| --- | --- | --- | --- |

1. If yes which rules (Please tick the box and give detailed information)

| □ | Maintenance | □ | Resource Allocation |
| --- | --- | --- | --- |
| □ | Operations | □ | Other (specify) |

1. Please list all types of benefits anybody enjoys related to the dam! (Please tick the box and give detailed information)

| □ | Agriculture^[[3]](#footnote-3)^ | □ | Domestic use |
| --- | --- | --- | --- |
| □ | Livestock | □ | Fishing |
| □ | Other (Specify) |  |  |

1. Last year, which individuals, groups or organizations received water from the dam? (Please name the households who used the dam water for irrigation.)

| Individuals, groups or organizations | Purpose of water use |
| --- | --- |
|  | □ *Agriculture* □ *Domestic* use □ *Livestock* □ *Fish* □ *Other (specify)* |
|  | □ *Agriculture* □ *Domestic use* □ *Livestock* □ *Fish* □ *Other (specify)* |
|  | □ *Agriculture* □ *Domestic use* □ *Livestock* □ *Fish* □ *Other (specify)* |
|  | □ *Agriculture* □ *Domestic use* □ *Livestock* □ *Fish* □ *Other (specify)* |
|  | □ *Agriculture* □ *Domestic use* □ *Livestock* □ *Fish* □ *Other (specify)* |
|  | □ *Agriculture* □ *Domestic use* □ *Livestock* □ *Fish* □ *Other (specify)* |
|  | □ *Agriculture* □ *Domestic use* □ *Livestock* □ *Fish* □ *Other (specify)* |
|  | □ *Agriculture* □ *Domestic use* □ *Livestock* □ *Fish* □ *Other (specify)* |
|  | □ *Agriculture* □ *Domestic use* □ *Livestock* □ *Fish* □ *Other (specify)* |
|  | □ *Agriculture* □ *Domestic use* □ *Livestock* □ *Fish* □ *Other (specify)* |
|  | □ *Agriculture* □ *Domestic use* □ *Livestock* □ *Fish* □ *Other (specify)* |
|  | □ *Agriculture* □ *Domestic use* □ *Livestock* □ *Fish* □ *Other (specify)* |
|  | □ *Agriculture* □ *Domestic use* □ *Livestock* □ *Fish* □ *Other (specify)* |

1. If the dam is not used for irrigation, could it potentially be used for irrigation^[[4]](#footnote-4)^?

| □ | Yes | □ | No |
| --- | --- | --- | --- |

1. Are there any rules or common practices related to the water extraction? (Please tick the box and give detailed information)

| □ | Maintenance | □ | Resource |
| --- | --- | --- | --- |
| □ | Operation | □ | Other (Specify) |

1. Have there been any conflicts around the dam? (Please tick the box and give detailed information)

| □ | Maintenance | □ | Resource allocation |
| --- | --- | --- | --- |
| □ | Operation | □ | Other (Specify) |

*In addition, it would be good if some engineer could give a rating of the dam condition and an estimate of the amount the specific dam can store when being well maintained (can be rough estimate).*

| *Capacity reduced due to siltation* | □ *Not* □ *very little* □ *little* □ *much* □ *very much* |
| --- | --- |
| *Capacity reduced due to overgrowing vegetation* | □ *Not* □ *very little* □ *little* □ *much* □ *very much* |
| *State of the earth walls* | □ *very good* □ good □ fair □ poor □ *very poor* |
| *State of the main wall* | □ *very good* □ good □ fair □ poor □ *very poor* |
| *State of the sluice gates* | □ *very good* □ good □ fair □ poor □ *very poor* |
| *State of the feeder channels* | □ *very good* □ good □ fair □ poor □ *very poor* |

Ask bellow questions only in those communities where game was played.

1. Has there been any change in the community regarding shared benefit from the common water resources after the exercise (game)?
2. Has the community come together for any kind of collective action (not limited to water resources) since the games was played?
3. Community members participated in the game discussed or share experience of the game with other community members/ in meeting/ in village council meeting?
4. After game played in the community, have community members prepared any group for taking care of the water resources?
5. In the community payment villages: How you utilized money received in the game?
6. In the individual payment villages: Some part of the money that you win was donated to the community, how you utilized that fund?

Supplementary Datasets

Dataset S1 (S1_roi.xlsx). Return on investment estimation for game intervention. This analysis builds on the check dam impact assessment by Action for Social advancement (ASA) from 2008.

Dataset S2 (S2_participant_characteristics.xslx). Game participants’ characteristics. The value displayed for t-tests are the differences in the means across the groups. The value displayed for F-tests are the F-statistics. ***, **, and * indicate significance at the 1, 5, and 10 percent critical level.

Dataset S3 (S3_balancing_test.xslx). Balancing test of earlier versus later villages. The value displayed for t-tests are the differences in the means across the groups. The value displayed for F-tests are the F-statistics. Standard deviations in parentheses. * indicates significance at the 10 percent critical level.

Dataset S4 (S4_village_characteristics.xslx). Overview of survey measures both at baseline and follow-up.

Dataset S5 (S5_main_SEM.xslx). Direct, indirect, and total effects from SEM for all leadership composition comparisons with “no leader” as the reference category. Estimates are from a model with robust standard errors in parentheses. * p<0.10, ** p<0.05, *** p<0.0

Dataset S6 (S6_GSEM.xslx). Robustness check with generalized SEM. Estimates are from generalized SEM model using multinomial logit estimates for leader composition, linear regression to estimate the share of female participants, probit link functions for the binary dam maintenance rule and maintenance outcomes with robust standard errors in brackets. * p<0.10, ** p<0.05, *** p<0.01

Dataset S7 (S7_SEM_robust.xslx). Robustness check: without baseline rule control. Estimates are from a model with robust standard errors in parentheses. * p<0.10, ** p<0.05, *** p<0.0

Dataset S8 (S8_intervention_effect.xslx). Intervention treatment effect relative to control villages. The dependent variable in models (1) to (2) is dam maintenance in the past 12 months (=1). Reported are the average marginal effects obtained after Probit regressions. The 95% confidence intervals with robust standard errors are reported in brackets: *** p<0.01, ** p<0.05, * p<0.1.

Dataset S9 (S9_leader_types.xslx). Different types of leaders that participated in the games.

Dataset S10 (S10_ingame_behavior_by_leader_composition.xslx). In-game differences in behavior and discussions depending on leader composition in the sessions.

SI References

1. M. A. Janssen, F. Bousquet, J.-C. Cardenas, D. Castillo, K. Worrapimphong, Field experiments on irrigation dilemmas. *Agricultural Systems* **109**, 65–75 (2012).

2. L. Bartels, T. Falk, V. Duche, B. Vollan, Experimental games in transdisciplinary research: The potential importance of individual payments. *Journal of Environmental Economics and Management* **113**, 102631 (2022).

1. Please make sure to use the name spellings consistent with those used in the previous baseline survey (please see sheet provided to you) [↑](#footnote-ref-1)
2. Please add community ID from sheet provided to you [↑](#footnote-ref-2)
3. If the dam is used for agriculture then skip question no 11 [↑](#footnote-ref-3)
4. If a dam is used for agriculture (question no 9)then skip this question [↑](#footnote-ref-4)
